# Molecular Pathology of Murine Ureteritis Causing Obstructive Uropathy with Hydronephrosis

**DOI:** 10.1371/journal.pone.0027783

**Published:** 2011-11-16

**Authors:** Osamu Ichii, Saori Otsuka, Yuka Namiki, Yoshiharu Hashimoto, Yasuhiro Kon

**Affiliations:** 1 Laboratory of Anatomy, Department of Biomedical Sciences, Graduate School of Veterinary Medicine, Hokkaido University, Sapporo, Japan; 2 Office for Faculty Development and Teaching Enriched Veterinary Medicine, Graduate School of Veterinary Medicine, Hokkaido University, Sapporo, Japan; Ulm University, Germany

## Abstract

Primary causes of urinary tract obstruction that induces urine retention and results in hydronephrosis include uroliths, inflammation, and tumors. In this study, we analyzed the molecular pathology of ureteritis causing hydronephrosis in laboratory rodents.

F2 progenies of C57BL/6 and DBA/2 mice were studied histopathologically and by comprehensive gene expression analysis of their ureters. Incidence of hydronephrosis was approximately 5% in F2 progenies. Histopathologically, this hydronephrosis was caused by stenosis of the proximal ureter, which showed fibrosis and papillary malformations of the proliferative epithelium with infiltrations of B-cell-dominated lymphocytes. Additionally, CD16-positive large granular leukocytes and eosinophils infiltrated from the ureteral mucosa to the muscular layer. Eosinophilic crystals were characteristically observed in the lumen of the ureter and the cytoplasm of large granular leukocytes, eosinophils, and transitional epithelial cells. Comprehensive gene profiling revealed remarkably elevated expression of genes associated with hyperimmune responses through activation of B cells in diseased ureters. Furthermore, diseased ureters showed dramatically higher gene expression of *chitinase 3-like 3*, known as *Ym1*, which is associated with formation both of adenomas in the transitional epithelium and of eosinophilic crystals in inflammatory conditions. The Ym1 protein was mainly localized to the cytoplasm of the transitional epithelium, infiltrated cells, and eosinophilic crystals in diseased ureters.

We determined that the primary cause of hydronephrosis in F2 mice was ureteritis mediated by the local hyperimmune response with malformation of the transitional epithelium. Our data provide a novel molecular pathogenesis for elucidating causes of aseptic inflammation in human upper urinary tracts.

## Introduction

In human clinical cases, aseptic inflammation in the upper urinary tracts, including the renal pelvis and the ureter, has been reported in diseases such as inflammatory pseudotumor (IPT) of the ureter, idiopathic segmental ureteritis (ISU), idiopathic retroperitoneal fibrosis involving the ureters (IRF), or eosinophilic ureteritis [1]–[5]. Symptoms such as fever, abdominal pain, and hematuria and detection of an obstructed urinary tract on contrast radiography suggest the presence of these diseases [1]. Obstruction of the upper urinary tract in these diseases induces urine retention in the renal pelvis, resulting in hydronephrosis and leading to end-stage renal failure. These cases are occasionally misdiagnosed as urinary stone disease or ureteric tumor because of their similar clinical symptoms [1], [2]. Furthermore, although uroliths in the upper urinary tract can be identified by a combination of urinalysis and radiography, differential diagnosis between tumor and inflammation is usually difficult in these portions [2]. Therefore, the definitive diagnosis and therapy of aseptic inflammation in the upper urinary tract must rely on histopathological examinations after nephroureterectomy [1]–[5]. To develop diagnostic methodology and novel therapy in addition to anti-inflammatory symptomatic treatments, elucidation of the molecular pathology of aseptic inflammation is required.

In experimental rodents, the pathologies of urinary tract obstructions have been analyzed in ureteral obstruction models by performing ureter ligations [6]. This experimental model is extremely useful for elucidating the pathology of renal tubulointerstitial lesions with hydronephrosis [6]. However, because cases of aseptic inflammation in the upper urinary tract are as scarce in laboratory animals as they are in human medicine, no good models have mimicked the human pathology of inflammatory ureteric obstructions. On the other hand, Marchesi *et al*. reported the spontaneous development of hydronephrosis due to ureteric obstructions caused by polypoid adenomas locating from the transitional epithelium of the renal pelvis to the ureter with severe cell infiltrations in promyelocytic leukemia (*PML)/*retinoic acid receptor, alpha (*RARα*) knock-in mice, a model for acute myeloid leukemia [7]. *PML/RARα*, the mutant gene causing leukemia in humans, results from a specific t(15;17) translocation that fuses *PML* and *RARα* [8]. Eosinophilic crystals containing a chitinase-like protein, which is associated with conditions of hyperplasia and inflammation, are a characteristic pathological feature of these mice and were detected in the cytoplasm of transitional epithelial cells (TECs) and neoplastic myeloid cells [7]. In addition, several clinical reports have showed chronic inflammatory infiltrations in the subepithelium of the urinary bladder and ureter in patients with systemic lupus erythematosus, and another report showed that eosinophilic ureteritis is usually observed in allergic patients [5], [9]. Therefore, these studies suggest that the molecular pathology of aseptic inflammation in the upper urinary tracts is strongly affected by changes in systemic or local immunological states such as myeloid leukemia, autoimmune disease, or allergic responses [1]–[5], [7]. Indeed, most cases of aseptic inflammation in the upper urinary tract are characterized by cellular infiltrations, sclerotic fibrosis, infiltration of eosinophils to the ureters, and additional common clinical symptoms [1]–[5].

In the present study, we report for the first time that the F2 progenies from inbred mice incidentally develop hydronephrosis subsequent to ureteritis with papillary malformations of the TECs and infiltration of lymphocytes and eosinophils. This pathological feature of ureteritis was characterized by the eosinophilic crystals in the cytoplasm of lymphocytes, eosinophils, and TECs. Furthermore, microarray analysis clearly revealed local hyperimmune conditions that were especially associated with the activation of B cells. Our data provide useful molecular pathological information about aseptic inflammation in the upper urinary tract and contribute to the development of targets for diagnosis of this idiopathic malignant disease.

## Results

### F2 progenies develop hydronephrosis

**Figure 1:**
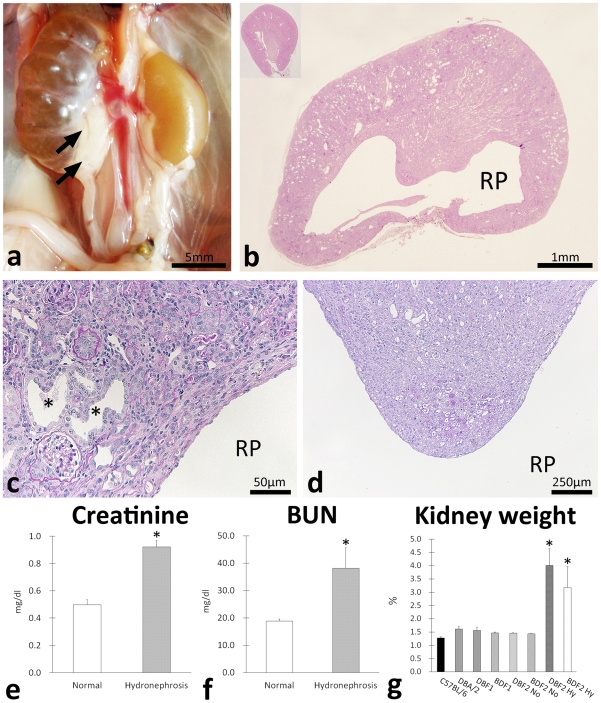
Clinicopathology of hydronephrosis in progenies of C57BL/6 and DBA/2. Gross anatomical features of hydronephrosis in F2 progenies of C57BL/6 and DBA/2 (BDF2) (panel a). Unilateral hydronephrosis showing urine retention to the renal pelvis was observed. Arrows indicate ureter hypertrophy in proximal portions. Kidney histological features in hydronephrosis (panel b). The renal cortex is pressed and thinned. RP: renal pelvis. C57BL/6 kidney is shown in the inset. Pressed renal cortex and medulla of BDF2 hydronephrosis kidney (panel c). Severe tubulointerstitial lesions such as dilations of renal tubular lumen, atrophies of renal tubules, interstitial cell infiltrations, and fibrosis were observed. Asterisks indicate dilated tubules. (panel d) Deep portion of renal medulla of BDF2 hydronephrosis kidney. Renal papilla of diseased kidney shows remarkable atrophy with dilated tubules, protein casts, and interstitial fibrosis. (panel e) Comparison of serum creatinine levels between normal and hydronephrosis F2 progenies. Values = mean ± S.E. *: significant differences between 2 groups (*p*<0.05). (panel f) Comparison of blood urea nitrogen (BUN) levels between normal and hydronephrosis F2 progenies. Values = mean ± S.E. *: significant differences between 2 groups (*p*<0.05). (panel g) Comparison of relative kidney weights among C57/BL/6, DBA/2, and their F1 and F2 progenies. Values = mean ± S.E. *: significant differences between normal and hydronephrosis F2 mice (Mann-Whitney *U* test, *p*<0.05). No: normal. Hy: hydronephrosis.

Necropsy revealed that only F2 progenies developed bilateral or unilateral hydronephrosis showing urine retention to the renal pelvis (Figure 1a). The incidence of hydronephrosis was approximately 5% in all F2 mice at 10 weeks and this incidence did not differ between the right and left kidney or between the sexes of the mice (Table 1). According to the severe dilations of the renal pelvis containing urine (Figure 1a), the diseased kidneys were pressed (Figure 1b). Severe tubulointerstitial lesions such as dilations of the renal tubular lumen, atrophy of the renal tubules, interstitial cell infiltrations, and fibrosis were observed in the cortices of diseased kidneys (Figure 1c). In addition, the renal papillae of diseased kidneys showed remarkable atrophy with dilated tubules, protein casts, and interstitial fibrosis (Figure 1d). Compared to normal mice, hydronephrotic F2 mice showed increased serum creatinine and serum blood urea nitrogen (BUN) levels, indicating deterioration of renal function (Figure 1e and f). The bilateral hydronephrotic F2 mice tended to show higher serum BUN levels than the unilateral ones (54.4±16.1 vs. 27.5±2.3, *P* = 0.09), but no differences were observed in the creatinine levels between these 2 groups. The relative kidney weight to body weight ratio of hydronephrotic mice was significantly higher than those of normal mice (Figure 1g).

**Table 1:** Incidence of hydronephrosis in progenies between C57BL/6 and DBA/2 mice.

Strain	Sex	Age (weeks)	Number of animals	Mice number of hydronephrosis*	Incidence of hydronephrosis
**DBF1**	Male	10–32	15	0	0.0
	Female	10–32	15	0	0.0
**BDF1**	Male	10–32	15	0	0.0
	Female	10–32	15	0	0.0
**DBF2**	Male	10	100	7	7.0
	Female	10	94	5	5.3
**BDF2**	Male	10	150	6	4.0
	Female	10	40	3	7.5
**F2 total**	-	10	384	21	5.5

DBF1: (DBA/2×C57BL/6)F1. BDF1: (C57BL/6×DBA/2)F1. DBF2: (DBF1×DBF1)F2. BDF2: (BDF1×BDF1)F2. *: Hydronephrosis is developed in either unilateral or both sides of kidneys. -: Not application.

### Ureteritis causes urinary tract obstruction

**Figure 2:**
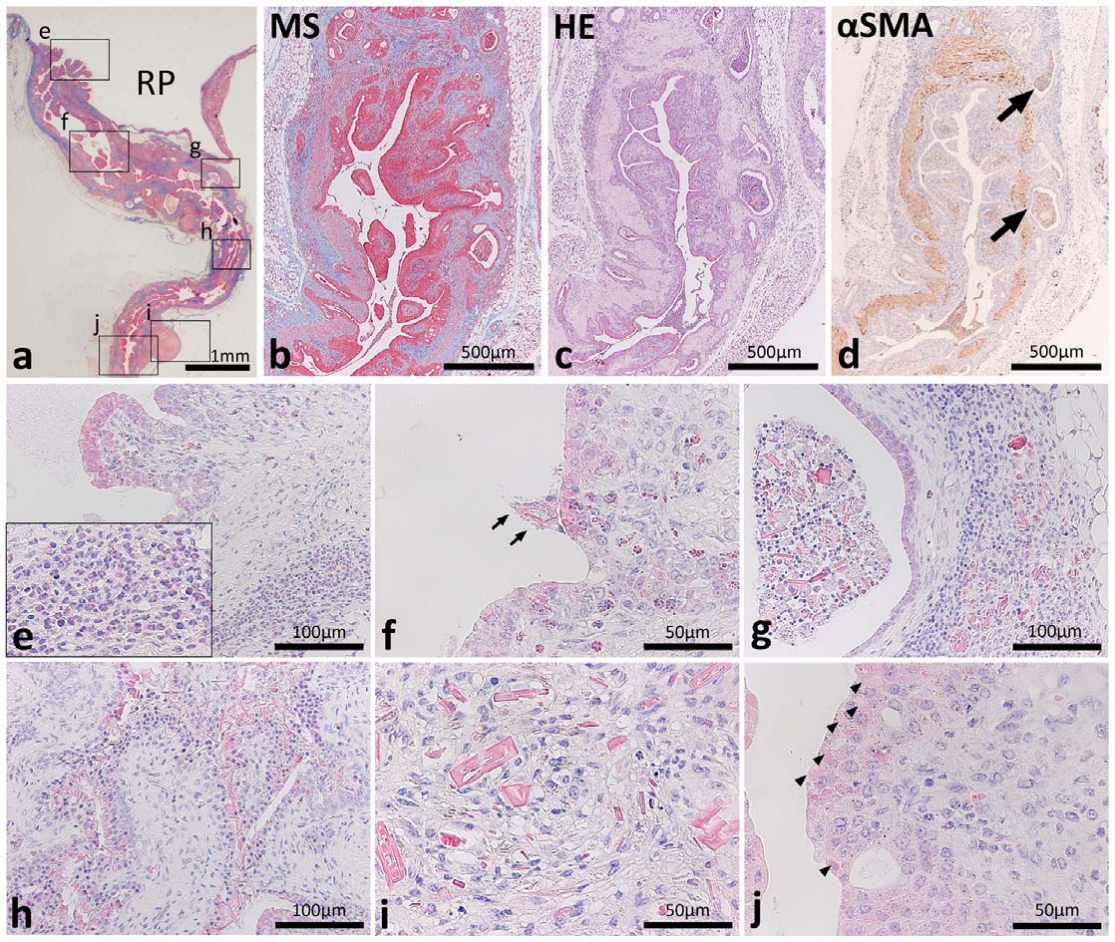
Histopathological features of ureteritis in F2 mice showing hydronephrosis. In panel a, letters and square areas indicate the observed areas of panels e–j in serial HE sections. BDF2 ureter hypertrophy was observed from the proximal portion (panel a). The ureter lumen showed stenosis featured by mucosa hypertrophy with fibrosis from the lamina propria to the muscular layer (panels b–d) and these lesions were more severe in the proximal portions of the ureter (panel a). Gland-like structures containing dead mononuclear cells, granulocytes, and dropped transitional epithelial cells were observed at the outside of the alpha smooth muscle actin (SMA)-positive muscular layer (panel d, arrows). Papillary malformations of the transitional epithelium were observed in the pelvic-ureteral junction (panels a and e) and eosinophil infiltrations were noted from the mucosa to the submucosa (panel e, inset). Large granular leukocytes infiltrated eosinophilic granules from the lamina propria to the transitional epithelium (panel f). Eosinophilic crystals and dropped cells were observed in the ureteral lumen (panel f, arrows), lesions of cell infiltrations (panel g), and transitional epithelium (panel h). Granuloma composed of mononuclear cells, granulocytes, and eosinophilic crystals was observed at the ureter adventitial position (panels a and i). Eosinophilic amorphous materials were observed in the apical cytoplasm of the transitional epithelium in the distal portions of the ureter (panel j, arrowheads). RP: renal pelvis. HE: hematoxylin and eosin stain. MT: Masson's trichrome stain. αSMA: immunohistochemistry.

Hydronephrotic F2 mice showed hypertrophy of the ureter from the proximal portion (Figure 2a). Histopathological analysis revealed papillary malformations of the transitional epithelium in the pelvic-ureteric junction (Figure 2a and e). The ureter lumen showed stenosis by hypertrophy of the mucosa with fibrosis and cell infiltrations from the lamina propria to the adventitia and these lesions were more severe in the proximal portions (Figure 2a). In most cases, gland-like structures were observed outside the muscular layer, and dead mononuclear cells, granulocytes, and dropped TECs were present in the lumens of the gland-like structures (Figure 2a–d, arrows). Furthermore, eosinophil infiltrations were observed in the proximal portions of the ureter (Figure 2e, inset). ­­In the ureter mucosa, large granular leukocytes with eosinophilic granules infiltrated from the lamina propria to the transitional epithelium (Figure 2f). Eosinophilic crystals and dropped cells were characteristically observed in the lumen of the diseased ureter (Figure 2f–h), lesions of cell infiltrations (Figure 2g), and the transitional epithelium (Figure 2h). In some cases, granuloma composed of mononuclear cells, granulocytes, and eosinophilic crystals was observed at the adventitial ureter position (Figure 2a and i). In the ureter distal portions, eosinophilic amorphous materials were observed in the apical cytoplasm of TECs and the cellular infiltrations and fibrosis were milder than in the proximal portions (Figure 2j).

### Cell infiltrations and epithelial proliferation in the diseased ureter

**Figure 3:**
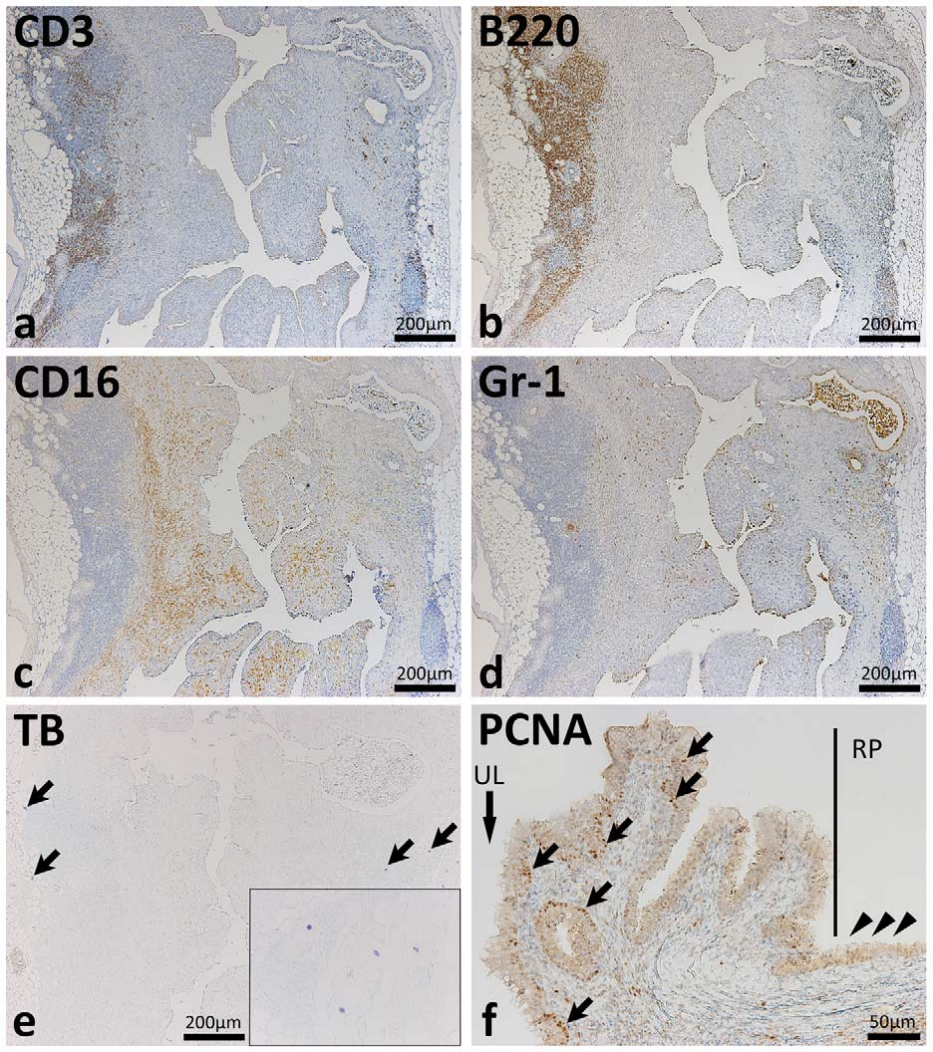
Inflammatory cell infiltrations and proliferation of the epithelium in the ureters of F2 mice showing hydronephrosis. Diseased ureters of BDF2 mice showed severe mononuclear cell infiltrations predominated by B220-positive cells (panel b). B220-positive cells and CD3-positive cells were mainly observed in the perivascular portions at the adventitia of ureters (panels a and b). CD16-positive cells were localized from the muscular layer to the lamina propria (panel c). Gr-1-positive cells were diffusely observed in the ureter and the lumen of gland-like structures (panel d). Metachromatic mast cells were not detected in the mucosa but several were observed in adipose tissue beside the adventitia (panel e, arrows and inset). In the pelvic-ureteral junction (panel f), proliferative cell nuclear antigen (PCNA)-positive cells were observed in the basal layer of the ureter transitional epithelium (arrows) but PCNA-positive nuclei were not detected in those of the renal pelvis (arrowheads). The black line indicates the border between the ureter and the renal pelvis (RP). UL: ureteral lumen. CD3, B220, CD16, Gr-1, PCNA: immunohistochemistry. TB: toluidine blue stain.

Immunohistochemistry targeting CD3 (T cells), B220 (B cells), CD16 (NK cells and activated macrophages), and Gr-1 (granulocytes) revealed severe inflammatory cell infiltrations in the diseased ureter (Figure 3a–d). The diseased ureter had numerous B220-positive cells (Figure 3b). B220-positive cells and CD3-positive cells were mainly observed in the perivascular portions at the adventitia (Figure 3a and b) whereas CD16-positive cells were observed from the muscular layer to the lamina propria (Figure 3c). Gr-1-positive granulocytes were diffusely observed in the ureter and in the lumen of gland-like structures (Figure 3d). No mast cells were observed in the mucus but several were located in the adipose tissue beside the adventitia (Figure 3e). In the pelvic-ureteral junction of hydronephrotic mice, PCNA-positive proliferative cells were observed in the basal layer of the ureteral transitional epithelium but PCNA-positive nuclei were not observed in the renal pelvis (Figure 3f).

### Ultrastructural pathology of ureteritis

**Figure 4:**
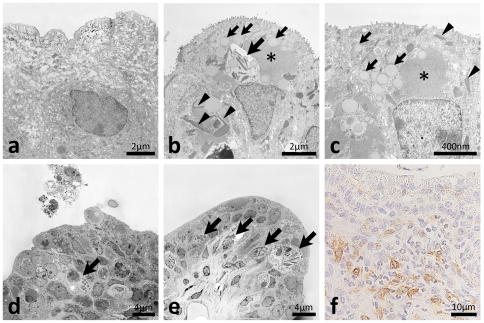
Ultrastructures of the ureteral mucus of F2 mice showing hydronephrosis. In the ureters of normal BDF2 mice, numerous fusiform vesicles were observed in the apical cytoplasm of transitional epithelial cells (panel a) whereas homogeneous, amorphous and round materials were observed in the apical of portions of the cytoplasm of diseased ureters (panels b and c, small arrows). Microvilli of transitional epithelial cells were more abundant in diseased ureters than in normal ureters (panels a–c). A high electron dense area was observed in the apical cytoplasm of transitional epithelial cells of diseased ureter (panels b and c, asterisks). Needle-shaped crystal-like materials were observed in the cytoplasms of transitional epithelial cells in diseased ureters (panels b and c, arrowheads). Mononuclear cells with nonsegmented nuclei and high electron dense materials infiltrated to the transitional epithelium and lamina propria of diseased ureters and some had needle-shaped crystal-like materials (panels b, d, and e, arrows). Some transitional epithelial cells and granulocytes with segmented nuclei and dense cores were observed in the ureter lumen (panel d). These granulated mononuclear cells showed positivity for CD16 by light microscopy (panel f). Malignant changes were not observed in the transitional epithelium of diseased ureters (panels b–e). Bars = nm, µm. CD16: immunohistochemistry.

In the diseased ureter, fusiform vesicles characterizing normal TECs were diminished (Figure 4a–c) and homogeneous, amorphous, and round materials were observed in the apical cytoplasm (Figure 4b and c). Clear malignant changes were not observed in the TECs of the diseased ureter (Figure 4b–e). The microvilli of TECs were more abundant in the diseased ureter than in the normal ureter (Figure 4b and c). Furthermore, a high electron dense area was observed in the apical cytoplasm of TECs in the diseased ureter (Figure 4b and c). The cytoplasm of TECs characteristically contained needle-shaped crystal-like materials in the diseased ureter (Figure 4b and c). Furthermore, mononuclear cells with nonsegmented nuclei and high electron dense materials infiltrated to the transitional epithelium and lamina propria of the diseased ureter and some had needle-shaped crystal-like materials similar to those of the TECs (Figure 4b, d, and e). Several TECs and granulocytes with segmented nuclei and dense cores were observed in the ureter lumen (Figure 4d). Light microscopy demonstrated positivity for CD16 in these granulated mononuclear cells (Figure 4f).

**Figure 5:**
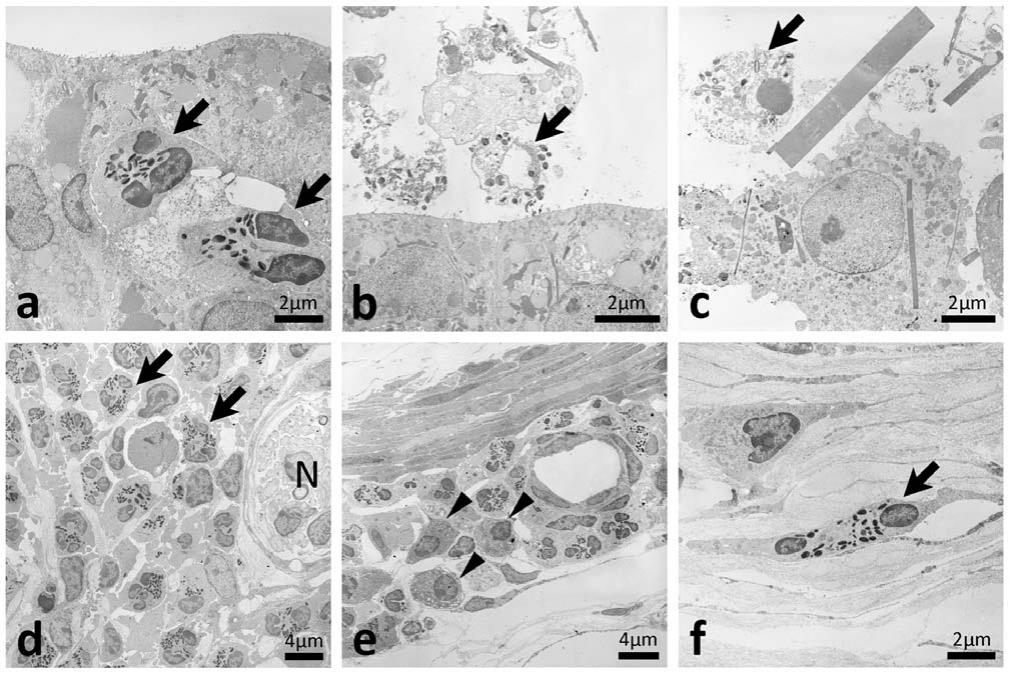
Eosinophil infiltrations and eosinophilic crystals in the ureters of F2 mice showing hydronephrosis. Eosinophils with segmented nuclei and dense core granules were observed in the transitional epithelium, lamina propria, and adventitia (panels a, d, e, and f, arrows). Cellular debris containing dense core granules resembling those of eosinophils and dropped epithelial cells containing dense core granules were observed in the ureter lumen (panels b and c, respectively). In adventitia, eosinophils were observed beside the nerve (panel d, N). Plasma cells with well-developed rough endoplasmic reticulum were observed with infiltrations of eosinophils adjacent to the muscular layer (panel e, arrowheads). Some eosinophils showed migrating patterns in the connective tissues (panel f).

In addition to granulated mononuclear cells, eosinophils with segmented nuclei and dense-cored granules were observed in the transitional epithelium, lamina propria, and adventitia (Figure 5a, d–f). Cell debris containing dense-cored granules resembling eosinophils and dropped epithelial cells containing dense-cored granules were observed in the ureter lumen (Figure 5b and c, respectively). Eosinophils were also observed in the adventitia beside nervous tissues (Figure 5d). Plasma cells were co-localized with perivascular infiltrations of eosinophils adjacent to the muscular layer (Figure 5e). Some eosinophils migrated into connective tissues (Figure 5f).

### Immune-associated genes in diseased ureters

The gene expression patterns of diseased ureters were comprehensively compared to those of the normal ureter (Tables 2 and 3). We performed Gene Ontology (GO) analysis on the list of significantly differentially expressed genes between the 2 groups and selected GO categories are listed with their Z scores in Table 2. Generally, genes associated with the immune response tended to show significant changes overall in expression in the diseased ureter, especially gene groups associated with B-cell functions such as B-cell activation, the B-cell receptor signaling pathway, B-cell proliferation, regulation of B-cell proliferation, positive regulation of B-cell proliferation, and the B-cell receptor complex (Table 2).

**Table 2:** Summary of results in microaray analysis between normal and diseased ureter.

Category	Subcategoly	Changed Genes	Total Genes	Z score	P-value
**Biological process**	immune system process	87	1132	9.66	1.35E-15
	response to wounding	53	539	9.65	1.07E-13
	immune response	51	524	9.37	4.53E-13
	cell activation	50	521	9.16	1.25E-12
	inflammatory response	38	340	9.14	1.19E-11
	B cell activation	25	176	8.98	5E-10
	propionate metabolic process	3	4	8.62	0.000703
	leukocyte activation	45	487	8.39	5.46E-11
	B cell receptor signaling pathway	8	28	8.13	0.00000549
	lymphocyte activation	40	423	8.08	3.51E-10
	renal system process involved in regulation of blood volume	5	12	8.03	0.0000794
	B cell proliferation	12	61	7.84	0.000000816
**Cellular component**	B cell receptor complex	5	10	8.85	0.0000426
	immunoglobulin complex	5	10	8.85	0.0000426
	extracellular region	118	1899	8.54	3.88E-14
	external side of plasma membrane	28	228	8.41	1.19E-09
	cell surface	41	423	8.29	1.51E-10
	immunoglobulin complex, circulating	4	8	7.92	0.000263
	sarcoglycan complex	4	8	7.92	0.000263
	mitochondrial membrane	39	449	7.27	7.29E-09
	mitochondrial envelope	40	470	7.21	7.93E-09
	dystroglycan complex	4	10	6.97	0.000509
	extracellular space	47	648	6.56	4.24E-08
**Molecular function**	antigen binding	12	26	13.12	3.28E-10
	chemokine activity	10	37	8.73	0.000000616
	chemokine receptor binding	10	37	8.73	0.000000616

Total probes: 35,587. Products: 36,142.

**Table 3:** Summary of microaray analysis data targeting genes expressed both normal and diseased ureter.

Rank	Normalized intensity (ratio vs normal)	GenBank Accession	Gene Symbol	Gene Name
**1**	119.6	J00544, NM_152839	*Igj*	immunoglobulin joining chain
**2**	104.7	U25103	*Igk-V32*	immunoglobulin kappa chain variable 32 (V32)
**3**	71.6	NM_008522	*Ltf*	Lactotransferrin
**4**	69.1	X63047	*Igh-VJ558*	immunoglobulin heavy chain (J558 family)
**5**	62.3	AI893574	*Igh-6*	immunoglobulin heavy chain 6 (heavy chain of IgM)
**6**	58.4	BQ947340	*Igl-V1*	immunoglobulin lambda chain, variable 1
**7**	58.3	NM_010810	*Mmp7*	matrix metallopeptidase 7
**8**	47.4	NM_008532	*Epcam*	epithelial cell adhesion molecule
**9**	45.4	NM_001024700	*Igh-VJ558*	immunoglobulin heavy chain (J558 family)
**10**	44.1	NM_009892	*Chi3l3*	chitinase 3-like 3
**11**	39.8	NM_007482	*Arg1*	arginase, liver
**12**	36.0	NM_008571	*Mcpt2*	mast cell protease 2
**13**	33.1	NM_010184	*Fcer1a*	Fc receptor, IgE, high affinity I, alpha polypeptide
**14**	29.5	NM_028968	*Ifitm7*	interferon induced transmembrane protein 7
**15**	25.8	NM_009402	*Pglyrp1*	peptidoglycan recognition protein 1
**16**	25.0	BC018315	*Igh-6*	immunoglobulin heavy chain 6 (heavy chain of IgM)
**17**	24.9	NM_146010	*Tspan8*	tetraspanin 8
**18**	24.8	NM_212451	*Ighg*	Immunoglobulin heavy chain (gamma polypeptide)
**19**	24.5	NM_008134	*Glycam1*	glycosylation dependent cell adhesion molecule 1
**20**	24.4	AK083315	*Ctse*	cathepsin E
**21**	23.9	NM_013605	*Muc1*	mucin 1, transmembrane
**22**	23.3	NM_021443	*Ccl8*	chemokine (C-C motif) ligand 8
**23**	21.3	NM_016958	*Krt14*	keratin 14
**24**	20.9	AY862185	*Serpina3g*	serine (or cysteine) peptidase inhibitor, clade A, member 3G
**25**	20.5	NM_011259	*Reg3a*	regenerating islet-derived 3 alpha

Total probes: 35,587. Products: 36,142.

Table 3 lists all genes that were elevated in the diseased ureter compared to the normal ureter. Genes associated with the construction of immunoglobulin chains were dramatically higher in the diseased ureter than in the normal ureter. In relation to the histopathology of the diseased ureter, the expression of genes associated with epithelium malformation (e.g., *Mmp7*, *Epcam*, *Tspan8,* and *Ctse*), the inflammatory response (e.g., *Ltf*, *Mmp7*, *Ifitm7*, *Glycam1*, *Ccl8, Arg1*, and *Chi3l3*), and the allergic response (e.g., *Mcpt2* and *Fcer1a*) were elevated in diseased ureters. Significant changes in *Pml* and *Rara* expression, the homologs of human *PML* and *RARα,* were not detected in the diseased ureter (data not shown, see GEO Series accession number GSE31098; http://www.ncbi.nlm.nih.gov/geo/query/acc.cgi).

### Localization of the Chi3l3/Ym1 protein

**Figure 6:**
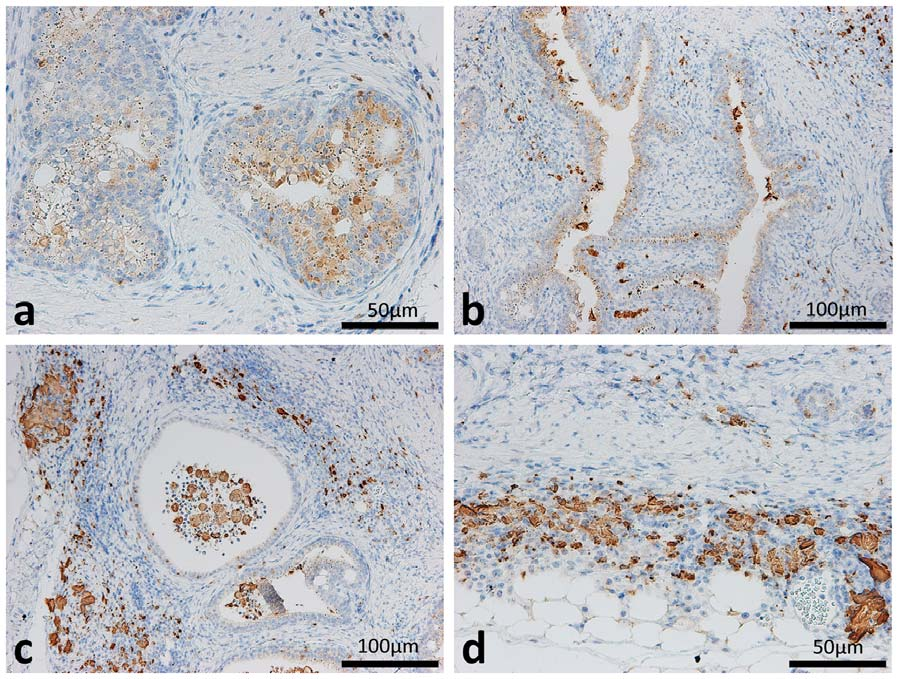
Distributions of Chi3l3/Ym1 protein in the ureters of F2 mice showing hydronephrosis. The Chi3l3/Ym1 protein was localized to the cytoplasm of transitional epithelium in the diseased ureter of BDF2 (panels a and b). Infiltrated cells in the lamina propria, dropped cells, and crystal structures in the lumen were immunohistochemically positive for Chi3l3/Ym1 (panels b and c). Infiltrated cells and crystal structures in adventitia beside adipose tissues were immunohistochemically positive for Chi3l3/Ym1 (panel d).

Based on the microarray results of the diseased ureter, we focused on elevated *Chi3l3* expression. The chitinase 3-like 3 (Chi3l3)/Ym1 protein is associated with both transitional epithelium adenoma and the formation of eosinophilic crystals [7]. As expected, the Chi3l3/Ym1 protein localized to the cytoplasm of TECs (Figure 6a and b) and infiltrated cells (Figure 6b–d) and eosinophilic crystals especially showed intense positive reactions (Figure 6a–d).

## Discussion

### Pathological type of ureteritis causing hydronephrosis

Urinary obstruction has been identified in approximately 10% of human renal failure patients [10], [11]. The major primary causes of urinary obstruction are tumors of the urinary tract or prostate and urinary stones, which can all be controlled by early diagnosis and adequate therapies [10], [11]. On the other hand, obstruction of the upper urinary tract caused by aseptic inflammation is clinically malignant and is the direct cause of hydronephrosis, and diagnosis and therapy must rely on biopsy and surgical dissections, respectively [1]–[5]. In the present study, we found incidental development of hydronephrosis with renal dysfunction in the F2 mice. Histopathologically, this hydronephrosis was directly caused by stenosis of the proximal ureters due to B-cell-dominated inflammations with fibrosis and proliferative ureteric malformations. The infiltrated CD16-positive cells were considered an NK cell or activated macrophage response to abnormal ureters. These inflammatory lesions characteristically contained eosinophils and granuloma was observed in several cases of F2 ureteritis. These pathological features overlap with aseptic inflammation in the upper urinary tract diagnosed as IPT of the ureter, ISU, IRF involving ureters, or eosinophilic ureteritis in humans [1]–[5]. The latter 2 diseases are accompanied by peritoneal fibrosis and systemic immunological changes such as atopy, respectively [2], [4], [5]. However, because these features were not observed in F2 mice with developing ureteritis, we suggest that F2 ureteritis resembles human IPT and ISU, which some researchers propose are the same entity [2]. The ureters in IPT and ISU show infiltrations of lymphoplasma cells and eosinophils and sclerotic fibrosis similar to F2 ureteritis [2]. Strikingly, some cases of IPT and ISU show ureteric malformations and granuloma, respectively [2], [3]. In experimental medicine, hydronephrosis is usually created by ureteric obstruction models by artificial ligation of the ureters [6]. Furthermore, the lesions of gene mutant models for hydronephrosis were restricted to the kidney [6]. Therefore, F2 mice are novel and useful models for hydronephrosis caused by aseptic inflammation in the upper urinary tract. We proposed that elucidating the molecular pathology of F2 ureteritis would provide fundamental information for clinically similar cases in humans.

### Relationship between ureteritis and genetic factors

Pathological features of F2 ureteritis were characterized by the appearance of eosinophilic crystals in the cytoplasm of TECs, inflammatory cells, and ureter lumens. In humans, eosinophilic crystals, which are called Charcot-Leyden crystals and are composed of endogenous lectin, were observed in several diseases with severe eosinophil infiltration such as parasitosis and asthma [12]. In experimental rodents, with the exceptions of parasitosis and infections, eosinophilic crystals were observed among organs showing immunological changes such as the lungs in eosinophilic macrophage pneumonia and the bile ducts under swine serum injections [13]–[15]. Interestingly, eosinophilic macrophage pneumonia with eosinophilic crystals is an idiopathic disease observed more frequently in mice with the C57BL/6 genetic background [13], [14]. Furthermore, most DBA/2 mice develop spontaneous eosinophilic myocarditis in the right ventricle characterized by calcinosis and eosinophil infiltration [16], [17]. Importantly, ureteritis was not observed in C57BL/6, DBA/2, or F1 progenies but was observed in the F2 progenies in this study. Therefore, these findings suggest that genetic backgrounds affect ureteritis development in mice. From this evidence, we concluded that the pathogenesis of ureteritis in F2 progenies was strongly affected by the summation of genetic interactions that mediate the susceptibility to immune-mediated diseases.

### What causes ureteritis?

Our microarray analysis results revealed remarkably elevated expression of genes associated with the inflammatory response (e.g., *Ltf*, *Mmp7*, *Ifitm7*, *Glycam1*, *Ccl8, Arg1*, and *Chi3l3*), allergic response (e.g., *Mcpt2* and *Fcer1a*), and epithelium malformation (e.g., *Mmp7*, *Epcam*, *Tspan8,* and *Ctse*). These data suggested local hyperimmune status of the diseased ureter. In a recent human clinical case, aseptic inflammation of the upper urinary tract resembling ISU, IPT, or IRF showed severe fibrosis and infiltration of plasma cells, lymphocytes, and eosinophils in the ureteral mass, and most of the plasma cells were IgG4-positive [18]. Interestingly, histopathological findings of the ureter in F2 mice were also characterized by infiltrations of lymphocytes and eosinophils, and the predominant infiltrations of activated B-cells were especially suggested by immunohistochemistry and GO analysis. Although the primary causes of ureteritis were unclear in this study, the local hyper immune response in the upper urinary tract due to factors (e.g., urinary substances) that stimulated IgG production and malformations of transitional epithelium was associated with the pathogenesis of F2 ureteritis.

In particular, the *Chil3l3* gene showed ectopic and remarkably elevated expression. The Chil3l3/Ym1 is an endogenous lectin without chitinase enzymatic activity that can bind to extracellular matrix glycosaminoglycans such as heparin/heparin sulfate [7], [19], [20]. In normal tissues, Chil3l3/Ym1 is widely distributed in mammalian bodies, suggesting its important roles in the immune response, differentiation of hematopoietic cells, and tissue remodeling [7], [19]–[21]. Chil3l3/Ym1 is expressed transiently in early myeloid precursor cells of hematopoietic tissues, showing initial expression in the yolk sac and subsequent expression in fetal liver, spleen, and bone marrow [20], [21]. Furthermore, Th2 cytokines associated with allergic responses induce Chil3l3/Ym1 expression in macrophages, suggesting the important role of Chil3l3/Ym1 in inflammation as well as hematopoiesis [22]. A previous study reported spontaneous development of hydronephrosis from ureteric obstructions caused by polypoid adenomas that localized from the renal pelvis to the ureter with severe cell infiltration and the appearance of Chil3l3/Ym1-positive crystals in acute myeloid leukemia of *PML/RARα* knock-in mice [7]. Although myeloid leukemia and changes in the expression of *Pml* and *Rara* were not observed in F2 progenies, the histopathological features of ureters from the *PML/RARα* knock-in mouse resemble those of F2 mice. In addition, Chil3l3/Ym1 was detected in the cytoplasm of the TECs, infiltrated cells, and eosinophilic crystals in F2 mice and these were also dropped in the luminal urine of the ureter. The chitinase family was recently suggested as a therapeutic target for the treatment of Th2 allergies and the usefulness of chitinase inhibitors has been reported [23], [24]. These findings indicate that Chil3l3/Ym1 would be a novel therapeutic target of aseptic inflammation of the upper urinary tract and the urinary detection of Chil3l3/Ym1 would serve as a non-invasive diagnostic method.

### Conclusion

We observed ureteritis with malformations of the transitional epithelium causing hydronephrosis in F2 progenies of C57BL/6 and DBA/2 mice and propose that this pathogenesis was based on altered local immunity through hyperimmune or allergic processes. Our results provide novel information regarding the molecular pathogenesis of aseptic inflammation of the upper urinary tract of humans.

## Materials and Methods

### Ethical statement

This study was approved by the Institutional Animal Care and Use Committee, which is convened at the Graduate School of Veterinary Medicine, Hokkaido University (approval number: 11-0030). The investigators adhered to the Guide for the Care and Use of Laboratory Animals of Hokkaido University, Graduate School of Veterinary Medicine (approved by the Association for the Assessment and Accreditation of Laboratory Animal Care International).

### Histopathology and serological analysis

Both sexes of C57BL/6 and DBA/2 mice were purchased from Japan SLC Inc. (Hamamatsu, Japan) and were maintained in specific pathogen-free conditions. F1 and F2 progenies were created from mating between these strains. The F1 mice generated by mating between female C57BL/6 and male DBA/2 were named BDF1 mice and those from reverse counter-partners were named DBF1 mice. F2 progenies were created by mating between the same F1 strains. All mice were sacrificed under deep anesthesia (pentobarbital sodium 60 mg/kg administered intraperitoneally) by exsanguination from the carotid arteries and the kidneys, ureters, and serum were immediately collected. The development of hydronephrosis was diagnosed by the retention of urine to the renal pelvis and gross anatomy kidney size. A section of each tissue sample was fixed in 4% paraformaldehyde at 4°C for histopathological analysis. From fixed tissues, paraffin-embedded sections of kidneys and ureters were stained with hematoxylin-eosin (HE), periodic acid Schiff (PAS), toluidine blue (TB), or Masson's trichrome (MT). The remaining fresh organ sections were stored in RNAlater solution (Ambion, Austin, TX, USA) for microarray analysis. To evaluate renal functions, serum BUN and creatinine levels were determined using the BUN-test-Wako and the Creatinine-test-Wako (Wako Pure Chemical Industries, Osaka, Japan), respectively.

### Transmission electron microscopy

For electron microscopy, a section of the ureter was fixed in 2.5% glutaraldehyde and 2% paraformaldehyde, postfixed in 1% osmium tetroxide, dehydrated in a graded alcohol series, and embedded in Quetol 812. Ultrathin sections (60 nm) were double stained with uranyl acetate and lead citrate.

### Immunohistochemistry

Immunohistochemistry for CD3, B220, Gr-1, alpha-smooth muscle actin (αSMA), proliferating cell nuclear antigen (PCNA), and chitinase 3-like 3 (Chi3l3/Ym1) in kidney paraffin sections was performed using unlabeled rabbit anti-CD3 antibody (Ab) (Nichirei, Tokyo, Japan), rat anti-B220 Ab (Cedarlane, Hornby, Ontario, Canada), rat anti-Gr-1 Ab (R&D Systems, Minneapolis, MN, USA), rabbit anti-αSMA Ab (Thermo Scientific, Waltham, MA, USA), mouse anti-human PCNA (Calbiochem, San Diego, CA, USA), and goat anti-mouse Chi3l3/Ym1 antibodies (R&D Systems), respectively. For antigen retrieval, the sections were autoclaved at 105°C for 15 min in 10 mM citrate buffer (pH 6.0) for αSMA and Dako Target Retrieval Solution (pH 9.0; Dako, Tokyo, Japan) for CD3, PCNA, and Chi3l3/Ym1. The sections were incubated with 0.1% pepsin/0.2 M HCl at 37°C for 5 min for B220 and Gr-1. We used 3,3′-diaminobenzidine (DAB) tetrahydrochloride-H_2_O_2_ solution for signal development.

### Microarray analysis

Normal ureters (n = 3) and diseased ureters (n = 3) were collected from BDF2 mice with developing unilateral hydronephrosis. The total RNA sample of each group was combined into 1 sample for analysis. The expression of 36,142 transcripts was profiled by an analysis service (Filgen, Nagoya, Japan). Briefly, RNA samples were checked for RNA integrity on a Bioanalyzer2100 (Agilent Technologies, Wilmington, DE, USA), labeled with Cy5, and hybridized to CodeLink™ Mouse Whole Genome Bioarray slides (Applied Microarrays, Tempe, AZ, USA). The slides were scanned using a GenePix 4000B laser scanner (Molecular Devices, Union City, CA, USA) and the images were digitized with CodeLink™ Expression Analysis v5.0 (Applied Microarrays). Data were normalized and are expressed as fold increase relative to data from the normal ureter using the MicroArray Data Analysis Tool Ver. 3.2 (Filgen). This MIAME-compliant dataset has been deposited in NCBI's Gene Expression Omnibus and is accessible through GEO Series accession number GSE31098 (approved Aug 02, 2011, http://www.ncbi.nlm.nih.gov/geo/query/acc.cgi).

### Statistical analysis

Results are expressed as the mean ± standard error (SE) and were statistically analyzed using the nonparametric Mann-Whitney *U* test (*p*<0.05).
